# Does Excess Tissue Sodium Storage Regulate Blood Pressure?

**DOI:** 10.1007/s11906-022-01180-x

**Published:** 2022-02-22

**Authors:** Giacomo Rossitto, Christian Delles

**Affiliations:** 1 Institute of Cardiovascular and Medical Sciences, BHF Glasgow Cardiovascular Research Centre, University of Glasgow, 126 University Place, Glasgow, G12 8TA UK; 2 Department of Medicine (DIMED), University of Padua, Padua, Italy

**Keywords:** Tissue sodium, Blood pressure, Hypertension, Volume, Blood vessels

## Abstract

**Purpose of Review:**

The regulation of blood pressure is conventionally conceptualised into the product of “circulating blood volume” and “vasoconstriction components”. Over the last few years, however, demonstration of tissue sodium storage challenged this dichotomous view.

**Recent Findings:**

We review the available evidence pertaining to this phenomenon and the early association made with blood pressure; we discuss open questions regarding its originally proposed hypertonic nature, recently challenged by the suggestion of a systemic, isotonic, water paralleled accumulation that mirrors absolute or relative extracellular volume expansion; we present the established and speculate on the putative implications of this extravascular sodium excess, in either volume-associated or -independent form, on blood pressure regulation; finally, we highlight the prevalence of high tissue sodium in cardiovascular, metabolic and inflammatory conditions other than hypertension.

**Summary:**

We conclude on approaches to reduce sodium excess and on the potential of emerging imaging technologies in hypertension and other conditions.

## Laragh’s Volume-Vasoconstriction Approach to Understanding Hypertension

A radically simple, “reductionist” view of the circulation would regard it as a system where a pump (heart) supplies a fluid (blood) via a tube (blood vessels) to the periphery (organs, tissues) [1]. In this model, Darcy’s law identifies blood flow (*Q*) as the difference in pressure across the system (Δ*P*, conveniently simplified to systemic arterial blood pressure given the trivial contribution of venous pressure to Δ*P* value) divided by resistance (*R*) [2]. Accordingly, blood pressure is determined by the product of the flow through the systemic circulation, i.e. cardiac output (including elements of volume and cardiac performance in its definition), and total peripheral resistance.

This equation underwent clinical conceptualisation by John Laragh who identified sodium (Na^+^), aldosterone and the kidneys contributing to “circulating blood volume”; and angiotensin and non-angiotensin vasoconstricting factors contributing to “vasoconstrictor components” [3, 4].

This concept provides a rational approach to contemporary antihypertensive therapies in the vast majority of patients, “as incontrovertible as the laws of physics” [5]. With the exception of the so-called decapitated hypertension, characterised by a reduction in blood pressure values independent of treatment and secondary to an overt reduction in ventricular function [6], the contribution of “cardiac performance” to the equation can generally be neglected and hypertension be treated with agents targeting its volume (mainly diuretics) or vasoconstriction (e.g. calcium channel blockers, ACE-inhibitors, AT_1_-receptor blockers) components, or a combination of them. In fact, current guidelines tend to favour early use of combination therapies that address more than one primary mechanism [1, 7] and reflect the multifactorial Mosaic view of hypertension pathogenesis, first proposed by Page [8] and recently reviewed and expanded by Harrison et al. [9]. Irrespective of current therapeutic pragmatism, the volume-vasoconstriction framework remains pathophysiologically valid, whereby the different components of the mosaic – no matter how complicated – would eventually impact one or the other aspect. Validity is such that polarisation of views [10] and overt controversy between renal and vascular “dysfunctionists” for the primacy of pathogenic mechanisms remain vivid [11, 12].

In the last few years, however, a novel aspect emerged that puzzlingly eludes Laragh’s dichotomous approach: the idea of tissue sodium storage. Na^+^, the leading player in the “volume” chapter of the story, has been suggested to accumulate in the extravascular compartment (i.e. out of the “tubes” and more precisely in the interstitial space, according to the original and ongoing conceptualisation) in animal models [13, 14, 15••] and patients with arterial hypertension [16, 17]. The question as to whether excess tissue sodium regulates blood pressure, as discussed in this review, naturally arises from this novel evidence.

## Description of Tissue Na^+^ Accumulation and Its Association with Hypertension

Although it was first suggested by Russian investigators in the 1970s [18], the concept of tissue Na^+^ was more fully developed by Jens Titze and co-workers over the last 20 years. In keeping with previous evidence of positive Na^+^ balance without commensurate water retention or body weight increases in subjects on short-term high salt diet [19], they reported a dissociation between Na^+^ and weight changes in unique long-term (months) observational [20] and interventional [21] studies, thus supporting the idea of a body reservoir for non-osmotic water-free sodium storage. Rat body-composition studies [14, 22–24] and ^23^Na-magnetic resonance imaging (MRI) in humans [16] pointed to the skin, and to some extent the muscle, as the specific depots for excess Na^+^ accumulation, with the negatively charged glycosaminoglycans (GAGs) network serving as the dynamically regulated substrate for putative interstitial skin Na^+^ binding [25].

The long-established association between salt and hypertension made hypertensive rodent models and patients with hypertension the most obvious targets to investigate the biological relevance of the phenomenon. Indeed, salt-sensitive rats exhibited excess skin Na^+^ retention upon salt-loading, paralleled by water [13, 22] but not to the point of iso-osmotically matching Na^+^ positive balance [14], and hypertensive patients with refractory hypertension or primary aldosteronism had increased tissue Na^+^ content compared with normotensive controls [17] or post-adrenalectomy follow-up [16], respectively. From these data, one can draw one first (#1) conclusion: hypertension, particularly, salt-sensitive hypertension, is associated with tissue Na^+^ accumulation.

## Osmotic and Non-osmotic Tissue Na^+^ in Hypertension

The first rodent studies suggested that a reduced osmotically inactive (i.e. water-independent) Na^+^ storage capacity upon salt-loading would confer salt-sensitivity of blood pressure via excess fluid retention [13]; if so, Na^+^ accumulation would be a beneficial buffering phenomenon to prevent volume excess. This idea was supported by a study in young (mean 30 ± 2, range: 18–49 years) healthy subjects randomised to a low Na^+^ diet (70 mmol/day) plus placebo or slow sodium (200 mmol/day) tablets for 7 days: skin Na^+^/K^+^ increased between placebo and slow sodium phases, although the change reached significance only in men and not women. The latter, at variance, experienced an increase in 24 h mean blood pressure (BP) [26].

Laffer et al. found salt-resistant (SR) and salt-sensitive (SS) normotensive subjects to exhibit similar Na^+^ balance during a protocol of salt-loading and depletion, but SR did not gain weight during Na^+^ retention whereas SS did [27]; remarkably, SS also failed to reduce their total peripheral resistance in response to salt loading (see below). An independent report recently suggested that healthy individuals could also store and “osmotically inactivate” significant amounts of sodium after hypertonic saline infusion [28]. While these studies lacked any direct assessment of skin water and electrolytes, these findings overall suggest that tissue Na^+^ buffering would reduce the adverse haemodynamic consequences of loading, i.e. (#2a) osmotic, but not non-osmotic, Na^+^ accumulation is associated with hypertension. Of course, association is not causation. Notably, ^23^Na-MRI skin signal did strongly and independently correlate with BP and left ventricular mass in patients with mild to moderate chronic kidney disease, but “similar associations were found for [MRI] skin water measurements” [29]. Is water the “bad” here, then?

In fact, results from seminal studies from the original proponents of the non-osmotic Na^+^ concept somehow challenged the above. In rats treated with high-salt diet, skin Na^+^ accumulation without reported commensurate water retention was paralleled by an increase in arterial BP. Inhibition of the VEGF-C signalling (induced by the tonicity-responsive enhancer binding protein [TonEBP] in interstitial mononuclear cells and shown to trigger expansion of the lymphatic network as a local Na^+^ draining system), as well as selective TonEBP deletion, augmented interstitial NaCl-to-water accumulation and elevated blood pressure in response to high-salt diet [23, 24]. Therefore, (#2b) lack of non-osmotic Na^+^ (and/or Cl^−^) clearance from tissues is associated with SS hypertension. Once again, in the absence of specific approaches targeting tissue-only Na^+^ excess, and ideally only its proposed non-osmotic component, association is not causation.

## A View Alternative to the Non-osmotic Compartment

The above conclusions on osmotic and non-osmotic interstitial Na^+^ may appear conflicting. Moreover, how does the proposed non-osmotic interstitial Na^+^ elude parallel water accrual while simultaneously driving TonEBP-mediated signalling? Notably, our recent body-composition study in SS and SR rats failed to confirm any hypertonic tissue Na^+^ accumulation [30••]. While tissue Na^+^ content and concentration did increase with high-salt diet, the findings were interpreted in light of a whole-tissue histochemical deductive approach [31–33] and appeared in keeping with a prediction model based on the extracellular-to-intracellular composition of tissues [34•]. In hypertensive patients, as in rats, skin Na^+^ excess was isotonic; tissue water, Na^+^ and Na^+^/K^+^ ratio were associated with pulse pressure, BNP, and, independent of any other covariate, with age [30••]. Consistently, a close correlation between human skin Na^+^ and water had already been independently reported [35]. Overall, the conclusions are that (#3) tissue Na^+^ excess is systemic and reflects extracellular volume (ECV) expansion compared to the intracellular (ICV), with or without an oedematous component. In fact, even the seminal studies reported that excess Na^+^ accumulation was accompanied by reduced ICV [23].

The proposition #3 brings the focus back to the volume extreme of the original dichotomic approach. Please note that ECV expansion does not equal intravascular volume expansion: upon salt-loading, after an “initiation” phase, the transient increase in cardiac output in both SS and SR subjects tends to resolve and cardiac output returns to normal or nearly normal values [36]. Moreover, there are subsets of hypertensive patients who have lower, rather than higher, plasma volume than normal subjects, and hypertensives as a group tend to have low plasma to interstitial fluid volume ratio, indicating that extracellular fluid distribution between the intravascular and interstitial compartments is largely shifted toward the latter [37–39].

On the other hand, why would ICV reduce? This may represent a relative reduction in face of an expanded ECV, but also active plasticity of the body cellular mass to accommodate (isotonic) excess Na^+^ while free water is excreted via renal or non-renal [40, 41, 42•] routes, as already suggested [43]; alternatively, it could be the passive result of a salt-induced catabolic state [44–46]. Both would point again to water, and not only Na^+^, handling as the core of the matter.

## A “Volume” Factor Impacting “Resistance”?

In the economy of physics of simple pipes, and of Laragh’s approach accordingly, neither a hypertonic nor a water-paralleled, but extravascular, Na^+^ excess appears to directly impact the cardiac output/intravascular volume side of the equation.

If a hypertonic accumulation in tissues is assumed, conclusion #2b would suggest a volume-independent effect of interstitial Na^+^ on arterial function. In cell culture experiments, supraphysiologic Na^+^ concentrations in the medium were shown to induce early changes in protein turnover and cellular hypertrophy of vascular smooth muscle cells [47]; however, ex vivo exposure of rat resistance vessels to hypertonic Na^+^ concentrations did not induce hypertensive hypercontractility or impaired relaxation [30••]. Still, lack of direct Na^+^-mediated vascular dysfunction does not exclude indirect effects: high Na^+^ has been independently shown to modulate the cells of the immune system and to induce a proinflammatory phenotype, as reviewed in detail elsewhere [48••, 49], which could locally impact vascular – and particularly endothelial [50, 51] – function. Whether any such hypertonic Na^+^ niche is actually encountered by immune cells in human pathophysiology remains to be proven, but even microscopic gradients (e.g. on the endothelial surface) [52•] in the absence of whole tissue hypertonicity could possibly suffice [43].

If one accepts the idea of the overall isotonicity of tissue Na^+^, instead, syllogisms are not easier: this “volume” would still be outwith the pipes. However, whether ECV expansion is due to extracellular matrix remodelling or to excess interstitial fluid, an impact on vascular function is conceivable. In the first case, hypertensive organ damage that includes changes in the interstitial matrix [53] and vascular fibrosis would per se result into excess ECV (and therefore tissue Na^+^ [34•]), would determine vascular stiffness and would raise BP accordingly. In the second case, with clinical or subclinical oedema, even microscopic excess of intercellular fluid in the vascular wall could disturb organ function, i.e. stiffen the vessels and increase total vascular resistance [54••, 55], without vasoconstriction sensu stricto. This contention, still awaiting confirmation, is in keeping with the hemodynamic characteristics of sodium-sensitive human subjects [27, 56], with the correlation between skin Na^+^/K^+^ ratio and peripheral vascular resistance [26] or pulse pressure [30••], as well as with functional impairment in the myocardium of hypertensive dogs [57, 58]. Moreover, accumulation of fluids even “outside” the vascular wall in the most peripheral interstitium would affect local biophysical homeostasis [59, 60] and possibly increase total vascular resistance by inducing at least functional, if not anatomical, microvascular and capillary rarefaction. Of note, many cell signalling pathways activated by dietary salt were previously described as “reminiscent of cell mechanoreceptor signalling” [50]: a not necessarily vascular volume expansion would nicely explain so.

**Fig. 1:**
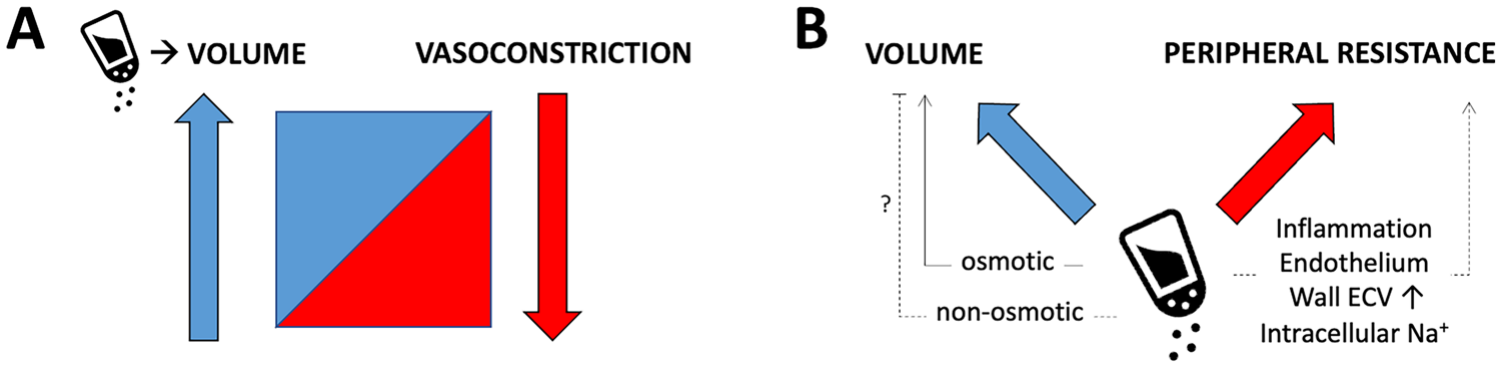
Sodium and regulation of blood pressure: classic and novel perspectives. **A** The vasoconstriction–volume spectrum of clinical hypertension (ref 4); Na^+^ (salt shaker) is considered the main determinant of the “intravascular volume” extreme of the spectrum, which variably combines with the independent “vasoconstriction” extreme to sustain different forms of high blood pressure (BP). **B** The current understanding of tissue Na^+^ accumulation expands beyond the vascular bed. Water-paralleled (osmotic) interstitial Na^+^, in equilibrium with the intravascular compartment, determines a whole-body volume excess; non-osmotic storage capacity could provide a buffering system which prevents a rise in BP, but lacks confirmation. Interstitial Na^+^ could simultaneously impact vascular function and increase peripheral resistance by inducing a local inflammatory state, endothelial damage, water-paralleled expansion of the extracellular volume (ECV) and changes in intracellular Na^+^ in the vascular wall

In summary, the idea of interstitial tissue Na^+^, either osmotic or non-osmotic, largely eludes a simplistic intravascular volume/vasoconstriction conceptualisation (Fig. 1) because (#4) human circulation is far from being a closed system of simple pipes.

## Would Targeting Tissue Na^+^ Improve Resistance?

An epidemiological association between altered arterial compliance and distensibility with high-salt diet, ultimately resulting in increased vascular resistance, has been suggested long ago [61]. However, more direct evidence in support of the above contentions should refer to tissue, rather than dietary, Na^+^ and to strategies that are known to reduce its accumulation. While enhancers of the lymphangiogenic response that provides interstitial drainage [23, 24] are still out of sight, different classes of natriuretic agents have consistently shown potential in this regard. Mineralocorticoid receptor antagonists (MRA) [16, 62•], loop diuretics [63], as well as the novel sodium-glucose co-transporter-2 (SGLT2) inhibitors [64] were all shown to reduce tissue Na^+^ content by means of ^23^Na-MRI.

Classic diuretics in the treatment of hypertension are often considered to have little or no effect on vascular health. Despite this assumption, thiazide and thiazide-like diuretics are in fact as effective as calcium channel blockers and angiotensin receptor blockers in reducing arterial stiffness and central pressure [65–67]. As these beneficial effects do not include improvements in endothelial function [68–70], a direct effect on vascular mechanics via reduction in tissue Na^+^ seems plausible. Of note, the secondary increase in plasma aldosterone upon diuretic treatment might in part counteract the effects of these drugs [71], thereby supporting the use of diuretics as part of a combination strategy with renin–angiotensin–aldosterone blockers [7].

MRAs even outperform other diuretics in improving arterial stiffness [72]. Their beneficial effects extend to endothelium-dependent flow-mediated vasodilatation [73], likely due to the role of MR in the modulation of endothelial stiffness and function [51, 74, 75•], and to highly specialised vascular beds such as coronary circulation [76, 77]. The novel nonsteroidal and highly selective finerenone, with recently demonstrated impact on hard cardiovascular outcomes in patients with chronic kidney disease and type 2 diabetes [78], seems to feature similar beneficial properties via structural remodelling of resistance vessels [79], potentially in keeping with the idea of “vascular wall congestion” [54••, 55].

SGLT2 inhibitors are novel antidiabetic agents that showed large and unexpected cardiovascular protection in multiple outcome trials, via mechanisms largely beyond glycaemic control and including early natriuresis [80]. Like the “cognate” natriuretic agents above, they were reported to reduce various parameters of arterial stiffness [81].

For the interpretation of all such evidence, particularly in relation to the pleiotropic effects of SGLT2 inhibition [80, 82], it is certainly hard to dissect the role of covariates, confounders and likely parallel mechanisms, including the distribution of excess sodium to the intracellular compartment and its consequences. However, it seems safe to state that (#5) agents with demonstrated potential to reduce tissue Na^+^ do exert beneficial effects on determinants of vascular resistance.

## Not Only Hypertension!

The remarkable expansion of ^23^Na-MRI data in the last few years has made very clear that, in addition to patient with hypertension, excess tissue Na^+^ signal is prevalent in a multitude of traditional cardiovascular risk factors and conditions, including heart failure [63] and chronic kidney disease [29]. After the first reports [64], patients with type-2 diabetes were recently shown to accumulate even greater skin and muscle Na^+^ than patients with primary hypertension [83•].

In addition, excess tissue Na^+^ signal was identified in the lower limbs of patients with lipedema [84•], a condition characterised by excess accumulation of adipocytes and inflammatory interstitial fluid [85, 86], in systemic sclerosis [87] and systemic lupus erythematosus [88]. Such evidence links the phenomenon to inflammation in general, without any direct implication on high blood pressure; this is also in keeping with high Na^+^ signal in the brain of patients with multiple sclerosis [89]. Whether all this reflects the extravasation of fluids typical of inflammation or a primary activation of inflammatory cells by local hypertonic niches [48••], or a combination of both, remains to be established. Of note, tissue changes with less inflammatory components but similar ECV expansion, like liver fibrosis, revealed the same increase in tissue Na^+^ signal [90, 91].

All in all, (#6) tissue Na^+^ accumulation appears to be a prevalent and systemic phenomenon which, as suggested, relates to acute or chronic changes in ECV distribution [34•] and – possibly – content. The implication of these changes may variably affect the different organs involved, including but not limited to blood vessels. The tremendous, although largely unexpected, beneficial effect that natriuretic agents recently showed in large cardiovascular outcome trials suggests cardiovascular implications beyond blood pressure control (43).

## Gaps of Knowledge and Conclusions

In summary, all the reviewed evidence above indicates that excess tissue Na^+^ accumulation contributes or at least relates to blood pressure control, but a radically simple “reductionist” approach to its understanding falls short in explaining much of this contribution. Laragh’s concept of “intravascular volume” did not include extravascular space, and a purely “vasoconstrictor component” definition disregarded aspects of vascular biology, structure and mechanics that are possibly affected by interstitial Na^+^ (Fig. 1).

Despite this awareness, we still face several “unknowns” in the field. The nature of the accumulation, hypertonic vs isotonic, is still debated. In case of hypertonic excess, evidence is conflicting in relation to a beneficial “buffering” function or a signature/determinant of disease, although the epidemic of high ^23^Na-MRI signal in cardiovascular and inflammatory diseases, as well as its association with organ damage, would suggest the latter. On the contrary, an isotonic (water-paralleled) ECV expansion could simultaneously be the driver and result of hypertensive hydraulic, inflammatory and fibrotic damage. An excess volume status seems indeed to accompany an increased tissue and total body sodium, but it is largely subclinical and often difficult to assess.

Of note, it is still unclear how Na^+^ is locally handled across vascular and cellular membranes and where exactly the excess accumulation develops, or why this happens in some individuals and not as much in others. Additionally, the fact that the large majority of body Na^+^ remains handled and excreted by the kidneys [92] leaves only a tiny signal for tissues and makes relevant investigations more challenging out of experimental settings. Similarly, the current lack of therapeutic tools targeting only interstitial Na^+^ excess prevents specificity of conclusions.

However, the beneficial impact of different natriuretic agents in hypertension and other cardiovascular conditions is now established and could be mediated at least in part by a reduction in tissue Na^+^ content. A wider use and the ongoing refinement of methods for its measurement [93] may facilitate better stratification of patients with hypertension, tailored reduction in salt intake and more targeted treatment.
